# Socio-demographic variation in stage at diagnosis of breast, bladder, colon, endometrial, lung, melanoma, prostate, rectal, renal and ovarian cancer in England and its population impact

**DOI:** 10.1038/s41416-021-01279-z

**Published:** 2021-02-09

**Authors:** M. E. Barclay, G. A. Abel, David. C. Greenberg, B. Rous, G. Lyratzopoulos

**Affiliations:** 1 The Healthcare Improvement Studies (THIS) Institute, Department of Public Health and Primary Care, University of Cambridge, Cambridge, UK; 2 Epidemiology of Cancer Healthcare and Outcomes (ECHO) Research Group, Department of Behavioural Science and Health, University College London, London, UK; 3 University of Exeter Medical School (Primary Care), Exeter, UK; 4 National Cancer Registration and Analysis Service, Public Health England, London, UK

**Keywords:** Oncology, Health policy

## Abstract

**Background:**

Stage at diagnosis strongly predicts cancer survival and understanding related inequalities could guide interventions.

**Methods:**

We analysed incident cases diagnosed with 10 solid tumours included in the UK government target of 75% of patients diagnosed in TNM stage I/II by 2028. We examined socio-demographic differences in diagnosis at stage III/IV vs. I/II. Multiple imputation was used for missing stage at diagnosis (9% of tumours).

**Results:**

Of the 202,001 cases, 57% were diagnosed in stage I/II (an absolute 18% ‘gap’ from the 75% target). The likelihood of diagnosis at stage III/IV increased in older age, though variably by cancer site, being strongest for prostate and endometrial cancer. Increasing level of deprivation was associated with advanced stage at diagnosis for all sites except lung and renal cancer. There were, inconsistent in direction, sex inequalities for four cancers. Eliminating socio-demographic inequalities would translate to 61% of patients with the 10 studied cancers being diagnosed at stage I/II, reducing the gap from target to 14%.

**Conclusions:**

Potential elimination of socio-demographic inequalities in stage at diagnosis would make a substantial, though partial, contribution to achieving stage shift targets. Earlier diagnosis strategies should additionally focus on the whole population and not only the high-risk socio-demographic groups.

## Background

Diagnosing patients at non-advanced stage is becoming a mainstay of contemporary cancer prevention and control strategies, complementing cancer prevention and screening.^1–4^ Most cancer patients are diagnosed after symptom onset, as effective screening tests only exist for few cancer sites and participation in screening programmes is suboptimal.^5^ Therefore, additional to efforts to optimise participation in screening, public health policies in many countries focus on shortening intervals from symptom onset to diagnosis to achieve population-level reductions in advanced stage cancer.

In 2018, the UK Government has set out a target for 75% of cancer patients with common solid tumours to be diagnosed in tumour, node, metastasis (TNM) stage I or II by 2028.^6^ However, how improvements in stage distribution could be achieved within a decade remains uncertain. An appealing strategy to help improve the stage distribution of incident cases is the reduction of stage inequalities by socio-demographic groups.

Several demographic and psychosocial factors are associated with the length of time from symptom onset to presentation (i.e. the ‘patient interval’^7^), and related markers (such as awareness of cancer symptoms or reported psychological or practical barriers to presentation).^8,9^ Specifically, older and lower socioeconomic status individuals tend to have lower awareness of likely cancer symptoms, higher degree of practical or emotional barriers to presentation and help-seeking and to experience longer intervals to diagnosis.^8,9^ Socio-demographic differences in stage at diagnosis are therefore likely even in populations served by healthcare systems without financial barriers to accessing care, though such associations may vary between different cancer sites.

Studies from various countries have documented socio-demographic variation in stage at diagnosis but chiefly focussed on the most common, ‘screenable’ cancers.^10–14^ Previous UK studies also tended to focus on regional, sub-national, populations and earlier eras.^15–18^ Motivated by the above considerations, we aimed to examine stage at diagnosis for common cancers in England and related socio-demographic variation in a recent study period with highly complete information on stage at diagnosis. We focussed on the cancer sites that have been used in public reporting of stage at diagnosis in local areas in England and that relate to the current UK government target for improving diagnosis of cancer at an earlier stage by 2028.^6^

## Methods

### Data

Data were analysed on incident cases aged 30–99 years diagnosed in 2015 with colon [International Classification of Diseases, Tenth Revision: C18], rectal [C19–C20], lung [C33–C34], melanoma [C43], (female) breast [C50], uterine [C54], ovarian [C56], prostate [C61], renal [C64], and bladder [C67] cancer registered in the English population-based cancer registry run by the Public Health England National Cancer Registration and Analysis Service. Beyond cancer site and integrated tumour TNM stage at diagnosis, the main analysis used information on the following variables: age (years), sex, small area deprivation group (fifth of the income domain of the Index of Multiple Deprivation 2015 score of the Lower Layer Super Output Area of the patient’s residence), screening detection status (for breast, colon, and rectal cancers only), and morphology group. Additional (auxiliary) variables were used in the imputation model as described below.

### Analysis

#### Parameterisation of stage at diagnosis

Consistent with existing reporting conventions in England and prior literature,^17–19^ in the main analysis we categorised stage at diagnosis as advanced/non-advanced using TNM stage III/IV and I/II, respectively. We explored alternative parameterisations in sensitivity analysis (see below).

#### Imputation of missing stage

Information on stage at diagnosis was 91% complete overall (range: 85% for ovarian to 94% for endometrial cancer). In our analysis, stage was the outcome variable; unlike the case for exposure variables, imputation of outcome variables is generally considered of limited value.^20^ However, if auxiliary variables are available, multiple imputation reduces bias and increases power compared with complete case analysis.^21^ Therefore, in the main analysis, we completed information on stage at diagnosis using multiple imputation by chained equations, separately for each cancer site. The imputation models included all variables used in main analysis (i.e. age, sex, deprivation group, screening detection status and morphology, parameterised as in main analysis) and several auxiliary variables, including survival (see Supplementary Information—Text Box). Ten imputations were produced for each site.

#### Statistical analysis

Initially, we described the number of patients in the data set by socio-demographic characteristic and cancer site and compared the observed and imputed proportions diagnosed at advanced stage by each variable.

We subsequently used three different logistic regression models with robust standard errors, with advanced/non-advanced stage categories being the binary outcome variable. First (Model 1) we described variation in advanced stage across the analysis sample (all ten studied cancer sites) adjusting for age at diagnosis (in years; modelled using a restricted cubic spline with knots at 40, 45, 55, 65, 75 and 85 years), sex, deprivation group and cancer site.

As associations between socio-demographic variables and stage at diagnosis could vary by cancer site, in a second model stratified by cancer site (Model 2) we described socio-demographic differences in stage at diagnosis (by age, sex and deprivation group) for each studied site separately.

For patients with breast, colon and rectal cancers, to acquire insights into the variation in stage at diagnosis that is mediated by socio-demographic differences in the proportion of screen-detected cases, we extended the stratified model also including screening detection status (Model 3).

For five of the ten studied cancers (lung, breast, renal, endometrial, ovarian), there is substantial morphological heterogeneity, and so all models were also adjusted for morphology group for these sites.

In sensitivity analysis, we examined alternative parameterisations of stage i.e. advanced stage defined as TNM II–IV or IV (in addition to III/IV in main analysis), restricting the analysis to those with recorded stage.

#### Estimating population-level impact

We estimated the population impact that would result from elimination of differences in stage at diagnosis by age (among patients aged ≥65 years), sex (for cancer other than female breast, prostate, ovarian and endometrial) and deprivation group. Specifically, we predicted the number of cases of advanced stage cancer we would expect if:

- Everyone aged >65 years had the same risk of advanced stage at diagnosis as those aged 65 years if the latter was lower.
- Men were to attain the same risk of advanced stage as women, or vice versa, as applicable for the sex with the lower risk.
- More deprived groups had the same risk of advanced stage at diagnosis as the least deprived group, if these groups had higher risk of advanced-stage cancer
- All above three socio-demographic differences were removed.

We applied the *mi predictnl* command in Stata to estimate the difference between the modelled probability of diagnosis at advanced stage and the probability of diagnosis at advanced stage under the counterfactual assumptions for each individual patient. We then summed these probabilities to estimate the total number of advanced stage diagnoses associated with each inequality.

## Results

Of the 202,001 incident tumours for the studied cancers diagnosed in 2015, 53% were diagnosed at stage I/II (‘non-advanced stage’), 38% at stage III/IV (‘advanced stage’), and 9% had missing stage (Table 1). After multiple imputation, 57.2 and 42.8% of tumours were diagnosed in non-advanced/advanced stage, respectively.

**Table 1:** Univariate observed and imputed stage distribution by variable category.

	Tumours	Observed						Imputed over 10 imputations)	
		Non-advanced stage	Advanced stage	Missing stage	% non-advanced	% advanced	% missing	% non-advanced	% advanced
Total	202,001	107,409	75,997	18,595	53.2%	37.6%	9.2%	57.2%	42.8%
Cancer									
Colon	23,452	9418	11,529	2505	40.2%	49.2%	10.7%	43.3%	56.7%
Rectal	11,279	4440	5884	955	39.4%	52.2%	8.5%	42.1%	57.9%
Lung	38,086	9203	25,841	3042	24.2%	67.8%	8.0%	25.8%	74.2%
Small cell	3807	210	3451	146	5.5%	90.6%	3.8%	5.7%	94.3%
Adenocarcinoma	11,946	3417	8138	391	28.6%	68.1%	3.3%	29.6%	70.4%
Squamous cell	7064	2113	4716	235	29.9%	66.8%	3.3%	30.5%	69.5%
Other non-small cell	3176	642	2429	105	20.2%	76.5%	3.3%	20.7%	79.3%
Specified other	844	424	285	135	50.2%	33.8%	16.0%	59.1%	40.9%
Unspecified	11,249	2397	6822	2030	21.3%	60.6%	18.0%	24.4%	75.6%
Melanoma	12,970	10,970	1080	920	84.6%	8.3%	7.1%	90.6%	9.4%
Breast^a^	45,432	35,861	6268	3303	78.9%	13.8%	7.3%	84.2%	15.8%
Infiltrating ductal carcinoma	35,116	28,703	4414	1999	81.7%	12.6%	5.7%	86.4%	13.6%
Lobular carcinoma	6036	4449	1167	420	73.7%	19.3%	7.0%	78.8%	21.2%
Mixed ductal lobular	921	719	162	40	78.1%	17.6%	4.3%	81.3%	18.7%
Other adenocarcinoma	2098	1634	260	204	77.9%	12.4%	9.7%	86.0%	14.0%
Other unspecified	1079	309	247	523	28.6%	22.9%	48.5%	43.5%	56.5%
Endometrial^a^	7316	5556	1334	426	75.9%	18.2%	5.8%	78.8%	21.2%
Adeno/endometrial low grade	4647	4118	373	156	88.6%	8.0%	3.4%	91.2%	8.8%
Adeno/endometrial high grade	1067	619	343	105	58.0%	32.1%	9.8%	61.1%	38.9%
Serous papillary	700	349	317	34	49.9%	45.3%	4.9%	51.8%	48.2%
Carcinosarcoma	370	202	153	15	54.6%	41.4%	4.1%	55.7%	44.3%
Clear cell	232	150	70	12	64.7%	30.2%	5.2%	66.9%	33.1%
Ovarian^a^	5002	1256	2996	750	25.1%	59.9%	15.0%	28.0%	72.0%
Serous high grade	2507	327	1921	259	13.0%	76.6%	10.3%	14.8%	85.2%
Carcinoma high grade	767	66	545	156	8.6%	71.1%	20.3%	9.7%	90.3%
Mucinous	268	217	34	17	81.0%	12.7%	6.3%	85.6%	14.4%
Unspecified/other	576	111	193	272	19.3%	33.5%	47.2%	29.0%	71.0%
Prostate	40,959	20,674	15,943	4342	50.5%	38.9%	10.6%	54.7%	45.3%
Renal	8933	4466	3297	1170	50.0%	36.9%	13.1%	56.8%	43.2%
Clear cell and papillary carcinoma	4623	2532	1736	355	54.8%	37.6%	7.7%	59.5%	40.5%
Renal cell carcinoma	3103	1458	1202	443	47.0%	38.7%	14.3%	54.2%	45.8%
Other and unspecified carcinoma	1207	476	359	372	39.4%	29.7%	30.8%	53.6%	46.4%
Bladder	8572	5565	1825	1182	64.9%	21.3%	13.8%	71.5%	28.5%
Adeno/SCC	385	152	184	49	39.5%	47.8%	12.7%	45.8%	54.2%
Non-papillary TCC	5060	3515	1082	463	69.5%	21.4%	9.2%	75.9%	24.1%
Papillary TCC	2153	1797	253	103	83.5%	11.8%	4.8%	87.3%	12.7%
Unspecified/other	974	101	306	567	10.4%	31.4%	58.2%	24.4%	75.6%
Sex									
Men	98,981	45,570	43,819	9592	46.0%	44.3%	9.7%	49.8%	50.2%
Women	103,020	61,839	32,178	9003	60.0%	31.2%	8.7%	64.2%	35.8%
Age, years									
30–39	3608	2474	875	259	68.6%	24.3%	7.2%	73.4%	26.6%
40–49	12,565	8638	3172	755	68.7%	25.2%	6.0%	72.8%	27.2%
50–59	28,358	17,908	8890	1560	63.1%	31.3%	5.5%	66.6%	33.4%
60–69	54,276	31,099	20,213	2964	57.3%	37.2%	5.5%	60.1%	39.9%
70–79	58,448	29,444	24,526	4478	50.4%	42.0%	7.7%	53.6%	46.4%
80–89	37,429	15,612	15,676	6141	41.7%	41.9%	16.4%	47.6%	52.4%
90–99	7317	2234	2645	2438	30.5%	36.1%	33.3%	41.2%	58.8%
Income deprivation quintile									
1—least deprived	43,401	24,636	14,670	4095	56.8%	33.8%	9.4%	61.4%	38.6%
2	44,946	24,884	15,953	4109	55.4%	35.5%	9.1%	59.6%	40.4%
3	41,661	22,329	15,516	3816	53.6%	37.2%	9.2%	57.4%	42.6%
4	37,685	19,061	15,074	3550	50.6%	40.0%	9.4%	54.4%	45.6%
5—most deprived	34,308	16,499	14,784	3025	48.1%	43.1%	8.8%	51.4%	48.6%
Screening status									
No or not applicable	186,007	93,520	74,315	18,172	50.3%	40.0%	9.8%	54.4%	45.6%
Yes	15,994	13,889	1682	423	86.8%	10.5%	2.6%	89.2%	10.8%

^a^Additional morphology groups not shown due to small numbers: Breast: other specified carcinoma and specified not carcinoma. Endometrial: sarcoma and unspecified/other. Ovarian: endometrioid, clear cell, carcinosarcoma, serous low grade, carcinoma low grade, and germ cell.

### Associations with advanced stage considering all the studied cancer sites together (Model 1)

In the unadjusted analysis, there was notable variation in advanced stage at diagnosis by sex (50 vs. 36% in men/women), age (27 vs. 59% in those aged 30–39/90–99 years) and deprivation group (39 vs. 49% in most/least deprived group patients). For lung, breast, ovarian, endometrial and bladder cancer, there were also substantial differences in stage at diagnosis by morphology type (Table 1). There was also large variation in the percentage of patients diagnosed at advanced stage by cancer site, ranging from lung (74%) and ovarian cancer (72%) to breast cancer (16%) and melanoma (9%).

**Fig. 1:**
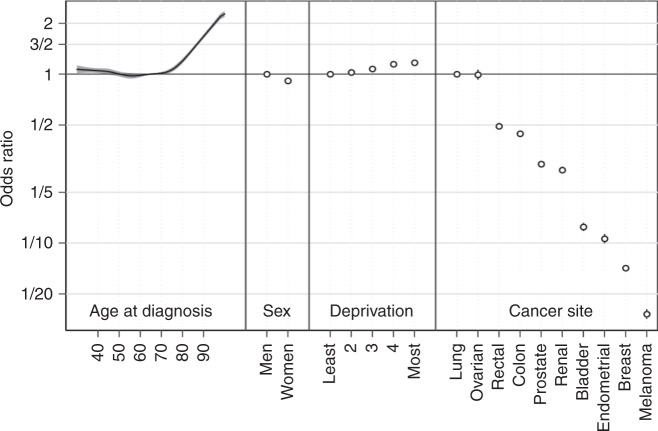
Adjusted odds ratios for diagnosis at advanced stage—all patients with any cancer site in the analysis sample (‘Model 1’ results). Confidence intervals are visualised if they are wider than the symbols used to show point estimates.

In the adjusted analysis, there was large variation in advanced stage at diagnosis in older age, with the odds increasing exponentially from 70 years upwards (Fig. 1). However, differences by sex and deprivation were relatively small. There was also very large variation by cancer site, which was substantially greater than that by age. For example, there was a 25-fold difference in the odds of advanced stage disease between lung cancer and melanoma (1/0.04), compared with up to 2-fold difference between those aged 95 and 65 years (1.88/1).

### Cancer site-specific adjusted associations with advanced stage at diagnosis (Model 2)

#### Age and stage

**Fig. 2:**
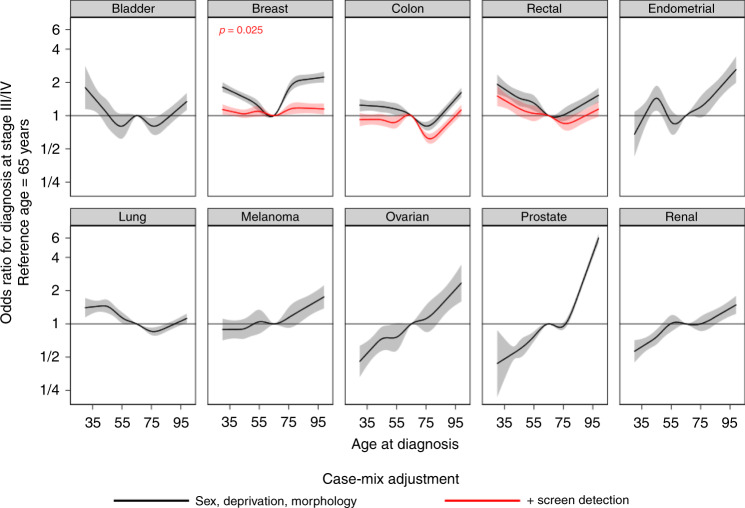
Adjusted odds ratios for diagnosis at advanced stage by age (30–99 years) from models stratified by cancer site (‘Model 2’ results). Unless otherwise reported, *p* < 0.001.

Age was strongly associated with risk of advanced stage disease for most cancer sites (*p* < 0.001, Fig. 2), with a monotonic increase in the odds of advanced stage at diagnosis with increasing age for 5 sites (melanoma, ovarian, prostate, renal and endometrial cancer), and a U-shaped association (both the younger and the older patients having relatively high odds of advanced stage) for the other 5 sites (breast, lung, colon, rectal, bladder cancer). For patients with breast, colon or rectal cancer, adjusting for screening detection status flattened differences by age in stage at diagnosis, particularly for breast cancer.

#### Sex and stage

**Fig. 3:**
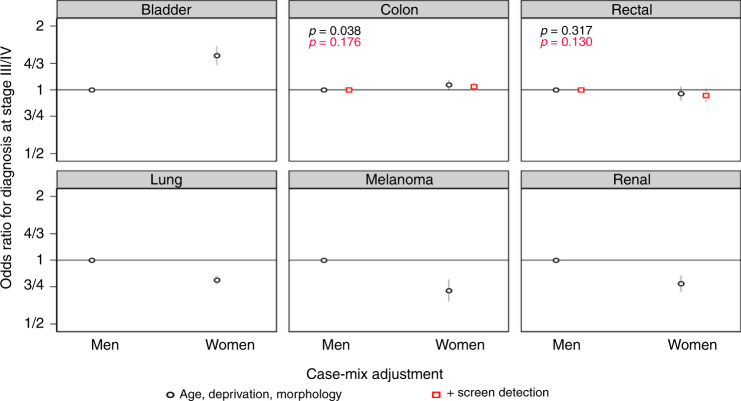
Adjusted odds ratios for diagnosis at advanced stage by sex from models stratified by cancer site (‘Model 2’ results). Where not reported, *p* < 0.001.

Across the 6 cancer sites that can occur in either sex, men were at higher risk of advanced stage at diagnosis for melanoma, lung and renal cancer, compared with women; in contrast, women had a higher risk of advanced stage at diagnosis of bladder cancer (Fig. 3). There was no evidence for variation in stage at diagnosis by sex for colon and rectal cancer, with or without adjustment for screening detection status.

#### Deprivation group and stage

**Fig. 4:**
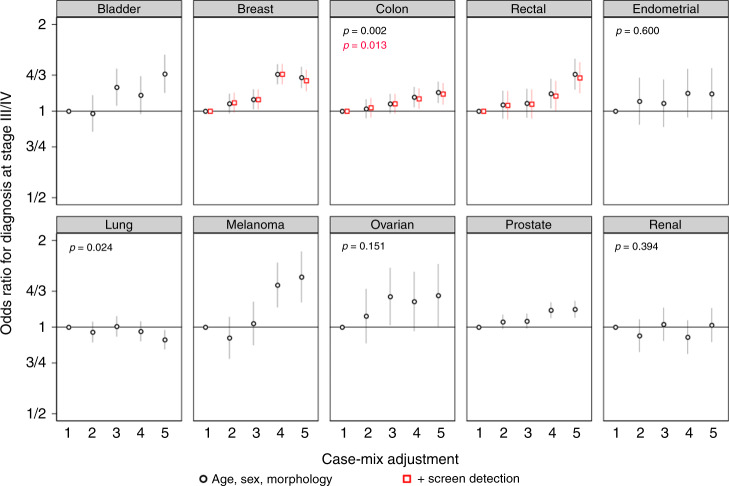
Adjusted odds ratios for diagnosis at advanced stage by income deprivation from models stratified by cancer site (‘Model 2’ results). Deprivation group 1 is the least deprived group. Unless otherwise reported, *p* < 0.001.

Increasing deprivation was associated with higher risk of advanced stage, with some variability by cancer (Fig. 4). For 7/10 sites (bladder, breast, colon, rectal, melanoma, ovarian and prostate cancer), more deprived patients were at higher risk of advanced stage at diagnosis. However, for lung, renal and endometrial cancer there was no evidence for differences in risk of advanced stage cancer between the deprivation groups (*p* > 0.05). For breast, colon and rectal cancer, additionally adjusting for screening detection status made little difference to associations with deprivation group. Values of data visualised in Figs. 1–4 are included Supplementary Information—Table 1.

### Population impact of removing deprivation and ‘older age’ inequalities in stage at diagnosis

**Fig. 5:**
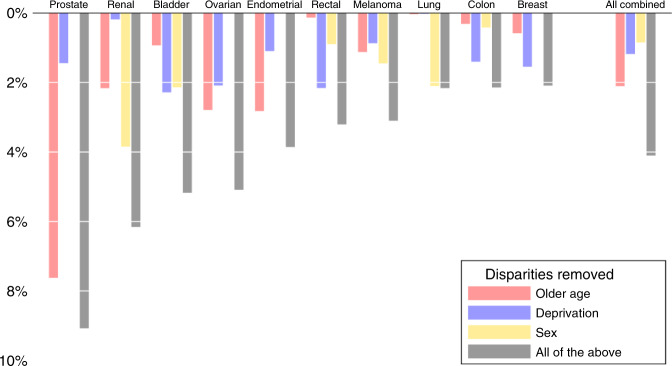
Estimated impact of removing ‘older age’ (among individuals ≥65 years), sex and income deprivation inequalities. Impact is shown as the reduction in the number of tumours diagnosed in stage III/IV as a percentage of total diagnoses of each cancer site, and of all sites combined.

For the studied cancers, removing socio-demographic inequalities in odds of advanced stage diagnosis would decrease the proportion of all incident cases diagnosed at advanced stage by 4.1%, or approximately 8,300 patients each year in England (Fig. 5 and Table 2—columns F and G). This would translate to 61.3% of patients with the 10 studied cancers being diagnosed at stage I/II, from the observed 57.2% (Table 3). Considering the absolute ‘distance’ to the aimed for 75% of all patients being diagnosed in stage I/II by 2028, such potential elimination would reduce the current ‘gap’ of 17.8% (75–57.2%) to 13.7%. And in relative terms, potential elimination would help cover 23.1% of the overall ‘distance to target’.

**Table 2:** Estimated impact of removing ‘older age’, sex and income deprivation inequalities, based on models adjusting for morphology and screening where appropriate.

Column A	Column B	Column C	Column D	Column E	Column F	Column G	Column H
Cancer	Impact of removing…	Total diagnoses	Stage III/IV cancers	(95% confidence interval)	Reduction in stage III/IV cases	(% of total diagnoses)	(% of cases diagnosed at stage III/IV)
All combined	Observed	202,001	86,435	(82,628, 90,242)			
	'Older age’ disparities		82,260	(78,468, 86,052)	4175	2.1%	5.1%
	Sex disparities		84,720	(80,914, 8855)	1715	0.8%	2.0%
	Deprivation disparities		84,152	(80,464, 87,841)	2283	1.2%	2.7%
	All 3 combined		78,148	(74,487, 81,808)	8287	4.1%	10.6%
Colon	Observed	23,452	13,267	(12,778, 13,756)			
	'Older age’ disparities		13,193	(12,705, 13,682)	74	0.3%	0.6%
	Sex disparities		13,169	(12,680, 13,658)	98	0.4%	0.7%
	Deprivation disparities		12,938	(12,457, 13,419)	329	1.4%	2.5%
	All 3 combined		12,764	(12,287, 13,241)	503	2.1%	3.9%
Rectal	Observed	11,279	6542	(6202, 6882)			
	'Older age’ disparities		6527	(6191, 6863)	15	0.1%	0.2%
	Sex disparities		6441	(6088, 6794)	101	0.9%	1.6%
	Deprivation disparities		6298	(5957, 6640)	244	2.2%	3.9%
	All 3 combined		6181	(5830, 6531)	361	3.2%	5.8%
Lung	Observed	38,086	28,256	(27,639, 28,873)			
	'Older age’ disparities		28,243	(27,629, 28,856)	13	0.0%	0.0%
	Sex disparities		27,455	(26,805, 28,106)	801	2.1%	2.9%
	Deprivation disparities		28,248	(27,622, 28,874)	8	0.0%	0.0%
	All 3 combined		27,433	(27,777, 28,089)	823	2.2%	3.0%
Melanoma	Observed	12,970	1232	(1020, 1444)			
	'Older age’ disparities		1086	(890, 1283)	146	1.1%	13.4%
	Sex disparities		1044	(855, 1232)	188	1.5%	18.0%
	Deprivation disparities		1119	(934, 1304)	113	0.9%	10.1%
	All 3 combined		830	(680, 981)	402	3.1%	48.4%
Breast	Observed	45,432	7136	(6577, 7695)			
	'Older age’ disparities		6871	(6314, 7429)	265	0.6%	3.9%
	Deprivation disparities		6433	(5919, 6946)	703	1.5%	10.9%
	Both combined		6186	(5677, 6695)	950	2.1%	15.4%
Endometrial	Observed	7316	1560	(1322, 1798)			
	'Older age’ disparities		1353	(1127, 1580)	207	2.8%	15.3%
	Deprivation disparities		1480	(1251, 1709)	80	1.1%	5.4%
	Both combined		1278	(1061, 1495)	282	3.9%	22.1%
Ovarian	Observed	5002	3602	(3392, 3812)			
	‘Older age’ disparities		3462	(3233, 3691)	140	2.8%	4.0%
	Deprivation disparities		3497	(3283, 3712)	104	2.1%	3.0%
	Both combined		3347	(3113, 3581)	254	5.1%	7.6%
Prostate	Observed	40,959	18,572	(18,013, 19,131)			
	'Older age’ disparities		15,449	(14,888, 16,011)	3123	7.6%	20.2%
	Deprivation disparities		17,981	(17,458, 18,504)	591	1.4%	3.3%
	Both combined		14,854	(14,334, 15,375)	3718	9.1%	25.0%
Renal	Observed	8933	3845	(3526, 4164)			
	'Older age’ disparities		3652	(3331, 3973)	193	2.2%	5.3%
	Sex disparities		3501	(3180, 3823)	344	3.9%	9.8%
	Deprivation disparities		3829	(3511, 4147)	16	0.2%	0.4%
	All 3 combined		3295	(2971, 3619)	550	6.2%	16.7%
Bladder	Observed	8572	2423	(2169, 2677)			
	'Older age’ disparities		2343	(2089, 2598)	80	0.9%	3.4%
	Sex disparities		2239	(2002, 2477)	184	2.1%	8.2%
	Deprivation disparities		2227	(1986, 2468)	196	2.3%	8.8%
	All 3 combined		1979	(1757, 2201)	444	5.2%	22.4%

**Table 3:** Percentage improvement in percentage of patients diagnosed in stage I/II by cancer site.

Cancer site	% stage I/II as observed	% stage I/II if socio-demographic differences are eliminated	Absolute increase in stage I/II (as % of all diagnoses)	Relative increase in stage I/II (as % of cases diagnosed in stage III/IV)
All combined	57.2%	61.3%	4.1%	10.6%
Prostate	54.7%	63.7%	9.1%	25.0%
Renal	56.8%	63.1%	6.2%	16.7%
Bladder	71.5%	76.9%	5.2%	22.4%
Ovarian	28.0%	33.1%	5.1%	7.6%
Endometrial	78.8%	82.5%	3.9%	22.1%
Rectal	42.1%	45.2%	3.2%	5.8%
Melanoma	90.6%	93.6%	3.1%	48.4%
Lung	25.8%	28.0%	2.2%	3.0%
Colon	43.3%	45.6%	2.1%	3.9%
Breast	84.2%	86.4%	2.1%	15.4%

In Table 2 (top, columns F and G), it can also be seen that potential elimination of inequalities in older age would decrease the proportion of all incident cases diagnosed in advanced stage by 2.1% (or around 4200 cases), whereas the corresponding figures for potential elimination of deprivation inequalities are 1.2% (or around 2300 cases) and for potential elimination of sex inequalities 0.8% (or around 1700 cases).

Considering the impact of potential elimination of socio-demographic inequalities in stage at diagnosis by cancer site, the largest absolute percentage increase in stage I/II was observed for prostate (9.1%) and the lowest for breast cancer (2.1%). Focussing on the two cancer sites with the greatest percentage of advanced stage cases (Table 1), for lung cancer removing socio-demographic inequalities would increase the percentage of cases diagnosed in stage I/II from 26% currently to 28%, while for ovarian cancer the respective percentage would increase from 28% currently to 33% (Table 3).

Within each specific cancer site, the relative the contribution of older age, deprivation and sex varied considerably. For example, for rectal cancer, elimination of deprivation inequalities alone would account for most of the potential reduction, compared to the contribution of eliminating age or sex inequalities. In contrast, for lung cancer potential elimination of sex inequalities would be most important (Table 2—column G). Values of data visualised in Fig. 5 are included Supplementary Information—Table 2. We also highlight the relative impact of potential elimination of inequalities in relation to reduction in advanced stage cases as a percentage of advanced stage diagnoses (Table 2—column H and Table 3, last column).

#### Sensitivity analysis

Complete case analysis provided smaller estimates of the excess risk of advanced stage cancer associated with older age, sex and deprivation (Supplementary Information—Figs. 1–3). In complete case analysis, alternative definitions of advanced stage categories gave similar results for sex and deprivation but different associations with age at diagnosis and cancer site (Supplementary Information—Figs. 4–6).

## Discussion

### Summary

We provide comprehensive evidence regarding socio-demographic disparities in stage at diagnosis of ten common cancers in a large European country population. Such associations tend to be present, but their strength and direction varies substantially by cancer site. Potential elimination of older age, deprivation and sex inequalities in stage at diagnosis would increase the percentage of patients diagnosed at stage I/II but the increase would fall short of the ‘75% of cases diagnosed in stage I/II’ target, though in proportional terms contributing nearly a quarter of the total improvement needed to attain it.

### Comparisons with prior literature

The findings substantially update and expand previous work limited to a single English region (East of England) and relating to an earlier study era (2006–2010).^18^ The larger (nationwide) sample size of the present study has improved estimate precision such that there is additional evidence of increasing risk of advanced stage at diagnosis in older age for ovarian, renal and lung cancer. Although we would not expect the findings to be necessarily concordant with those from other country populations, in general they are in keeping with literature documenting the presence of a variable degree of socio-demographic variation in stage at diagnosis for few common cancers.^10–16^ However, compared with most previous studies, we consider a larger number of common and rarer cancers together, take into account screening detection status and tumour morphology and estimate the population impact of inequalities in stage at diagnosis. Across our analysis sample, cancer site was associated with the largest amount of variation in stage at diagnosis, which would support accounting for cancer site case-mix in summary indicators of cancer stage at diagnosis for geographically defined populations. Regarding lung cancer in particular, we did not observe an association between stage at diagnosis and deprivation; this is consistent with other evidence and a meta-analysis of the global literature on socioeconomic status and stage at diagnosis of lung cancer.^22–24^

### Strengths and limitations

A strength of our study is that we were able to adjust for screening detection status, although this adjustment would not take into account any indirect impact of screening on non-screening detected cases. We found that adjustment for screening detection status mediates associations with older age substantially (as can be expected, as screening is targeted to specific age groups) but has a relatively limited impact on differences in stage at diagnosis by deprivation. A further strength is that we have adjusted for tumour morphology differences. We could not adjust for use of prostate-specific antigen (PSA) and some of the socio-demographic variation in advanced stage at diagnosis of prostate cancer may reflect greater use of PSA testing in the least deprived groups.^25^ As our study was motivated by the public health target for attaining earlier stage diagnosis for 75% of cancer patients by 2028, we did not consider the translation of differences in stage at diagnosis into differences in life expectancy and number of years lost due to inequalities. Eliminating differences in stage at diagnosis between different age groups and cancer sites will translate to variable impact in life years gained (depending on the age and cancer site case-mix of the cancers that will be diagnosed at an earlier stage). These questions should be addressed by future research.

There were inconsistent differences in stage at diagnosis by sex, with women being at greater risk of advanced stage than men for bladder cancer and at lower risk for lung, melanoma and renal cancer, with minimal variation for colon and rectal cancer. These heterogeneous patterns of sex differences in stage at diagnosis point to variable, cancer site-specific aetiologies. For example, prior research indicates that lung tumours tend to grow faster in men than women—consistent with the pattern we observed.^26^ Social factors may contribute to sex differences in stage at diagnosis for melanoma, such as differences between men and women in bodily awareness and help-seeking behaviour. Lastly, healthcare-related factors such as prolonged intervals from presentation to referral in women may be at least partly responsible for sex differences in stage at diagnosis of bladder cancer.^27,28^

Stage data were highly complete overall, though, as is the case for studies using nationwide population-based registry data, a small minority of patients had missing stage information. Concordant with best practice in this field, we used multiple imputation to assign stage (using information from several auxiliary variables included in the imputation but not the analysis model), which mitigates this limitation.^29–32^ Complete case analysis provided very similar findings.

Consistent with the public policy target that our research was motivated by, we have not considered the potential impact of elimination of variation in stage at diagnosis onto inequalities in survival or life years gained by the patient group.^33^

### Implications

The findings suggest the presence of common reasons for differences in stage at diagnosis among older people and between deprivation groups, across cancer sites. These may include psychosocial factors acting as barriers to prompt presentation/help-seeking.^34,35^ Targeted public health awareness campaigns focussing on specific cancers and/or population groups at higher risk of advanced stage disease would therefore be justified given the findings, particularly for cancer sites with a symptom signature dominated by symptoms with relatively high positive predictive value, for example for melanoma (melanotic skin lesion), breast (breast lump), bladder (haematuria), rectal (rectal bleeding) and endometrial cancer (post-menopausal bleeding).^36^ The majority of cancer patients with these symptoms have non-advanced stage disease.^37^

Although deprivation is not associated with variation in stage at diagnosis of lung cancer, given the very strong socioeconomic gradients in incidence, preventive efforts (e.g. through smoking cessation policies) are strongly justified. Increasing participation in bowel cancer screening can also help to achieve a favourable earlier stage shift.

In spite of clear socio-demographic inequalities in stage at diagnosis, their potential reduction would contribute substantially to achieving earlier stage diagnosis targets but will only help ‘bridge’ around a quarter of ‘distance-to-target’. Therefore, public health strategies to improve the distribution of stage at diagnosis of cancer should additionally focus on the whole population, rather than socio-demographic groups at higher risk. Novel diagnostic tests and strategies are additionally needed to enable earlier detection of cancer in both asymptomatic and symptomatic patients of any socio-demographic group.^38^

## Supplementary information

Supplementary online material

## Data Availability

The de-personalised data used in this study can be made available through application to Public Health England’s Office for Data Release.

## References

[1] World Health Organisation. *Guide to Cancer Early Diagnosis* (World Health Organization, 2017).

[2] Independent Cancer Taskforce. Report of the Independent Cancer Taskforce. Achieving world-class cancer outcomes - a strategy for England 2015-2020. https://www.cancerresearchuk.org/sites/default/files/achieving_world-class_cancer_outcomes_-_a_strategy_for_england_2015-2020.pdf (2016).

[3] CDC. Get the facts about gynecological cancer. https://www.cdc.gov/cancer/knowledge/publications/brochures.htm (2017).

[4] Danish National Board of Health. National Cancer Plan II: National Board of Health recommendations for improving cancer healthcare services (Copenhagen). http://www.sst.dk/~/media/A7052DCF93A641508A48A5B60A933A7D.ashx (2005).

[5] Elliss-Brookes L, McPhail S, Ives A, Greenslade M, Shelton J, Hiom S (2012). Routes to diagnosis for cancer - determining the patient journey using multiple routine data sets. Br. J. Cancer.

[6] UK Government. Government announces plans for earlier diagnosis for cancer patients. https://www.gov.uk/government/news/government-announces-plans-for-earlier-diagnosis-for-cancer-patients (2018).

[7] Weller D, Vedsted P, Rubin G, Walter FM, Emery J, Scott S (2012). The Aarhus statement: improving design and reporting of studies on early cancer diagnosis. Br. J. Cancer.

[8] Robb K, Stubbings S, Ramirez A, Macleod U, Austoker J, Waller J (2009). Public awareness of cancer in Britain: a population-based survey of adults. Br. J. Cancer.

[9] McCutchan GM, Wood F, Edwards A, Richards R, Brain KE (2015). Influences of cancer symptom knowledge, beliefs and barriers on cancer symptom presentation in relation to socioeconomic deprivation: a systematic review. BMC Cancer.

[10] Feller A, Schmidlin K, Bordoni A, Bouchardy C, Bulliard JL, Camey B (2018). Socioeconomic and demographic inequalities in stage at diagnosis and survival among colorectal cancer patients: evidence from a Swiss population-based study. Cancer Med..

[11] Booth CM, Li G, Zhang‐Salomons J, Mackillop WJ (2010). The impact of socioeconomic status on stage of cancer at diagnosis and survival: a population‐based study in Ontario, Canada. Cancer.

[12] Schwartz KL, Crossley-May H, Vigneau FD, Brown K, Banerjee M (2003). Race, socioeconomic status and stage at diagnosis for five common malignancies. Cancer Causes Control.

[13] Clegg LX, Reichman ME, Miller BA, Hankey BF, Singh GK, Lin YD (2009). Impact of socioeconomic status on cancer incidence and stage at diagnosis: selected findings from the surveillance, epidemiology, and end results: National Longitudinal Mortality Study. Cancer Causes Control.

[14] Kweon SS, Kim MG, Kang MR, Shin MH, Choi JS (2017). Difference of stage at cancer diagnosis by socioeconomic status for four target cancers of the National Cancer Screening Program in Korea: results from the Gwangju and Jeonnam cancer registries. J. Epidemiol..

[15] Brewster DH, Thomson CS, Hole DJ, Black RJ, Stroner PL, Gillis CR (2001). Relation between socioeconomic status and tumour stage in patients with breast, colorectal, ovarian, and lung cancer: results from four national, population based studies. BMJ.

[16] Adams J, White M, Forman D (2004). Are there socioeconomic gradients in stage and grade of breast cancer at diagnosis? Cross sectional analysis of UK cancer registry data. BMJ.

[17] Lyratzopoulos G, Abel GA, Barbiere JM, Brown CH, Rous BA, Greenberg DC (2012). Variation in advanced stage at diagnosis of lung and female breast cancer in an English region 2006-2009. Br. J. Cancer.

[18] Lyratzopoulos G, Abel GA, Brown CH, Rous BA, Vernon SA, Roland M (2013). Socio-demographic inequalities in stage of cancer diagnosis: evidence from patients with female breast, lung, colon, rectal, prostate, renal, bladder, melanoma, ovarian and endometrial cancer. Ann. Oncol..

[19] NHS England. CCG Outcomes Indicator Set 2014/15: technical guidance. https://www.england.nhs.uk/south/wp-content/uploads/sites/6/2017/07/ccg-outcomes-indicator-set-tech-guidance.pdf (2013).

[20] Von Hippel PT (2007). Regression with missing Ys: an improved strategy for analyzing multiply imputed data. Socio. Methodol..

[21] White, I. R., Royston, P. & Wood, A. M. Multiple imputation using chained equations: issues and guidance for practice. *Stat. Med*. **30**, 377–399 (2011). 10.1002/sim.4067 21225900

[22] Barclay ME, Abel GA, Elliss-Brookes L, Greenberg DC, Lyratzopoulos G (2019). The influence of patient case-mix on public health area statistics for cancer stage at diagnosis: a cross-sectional study. Eur. J. Public Health.

[23] Cheyne L, Taylor A, Milton R, Fear J, Callister ME (2013). Social deprivation does not affect lung cancer stage at presentation or disease outcome. Lung Cancer.

[24] Forrest LF, Sowden S, Rubin G, White M, Adams J (2017). Socio-economic inequalities in stage at diagnosis, and in time intervals on the lung cancer pathway from first symptom to treatment: systematic review and meta-analysis. Thorax.

[25] Moss S, Melia J, Sutton J, Mathews C, Kirby M (2016). Prostate-specific antigen testing rates and referral patterns from general practice data in England. Int. J. Clin. Pract..

[26] Ten Haaf K, van Rosmalen J, de Koning HJ (2015). Lung cancer detectability by test, histology, stage, and gender: estimates from the NLST and the PLCO trials. Cancer Epidemiol. Biomark. Prev..

[27] Lyratzopoulos, G., Abel, G. A., McPhail, S., Neal, R. D. & Rubin, G. P. Gender inequalities in the promptness of diagnosis of bladder and renal cancer after symptomatic presentation: evidence from secondary analysis of an English primary care audit survey. *BMJ Open* **3**, e002861 (2013). 10.1136/bmjopen-2013-002861 PMC3693425 23798742

[28] Dobruch J, Daneshmand S, Fisch M, Lotan Y, Noon AP, Resnick MJ (2016). Gender and bladder cancer: a collaborative review of etiology, biology, and outcomes. Eur. Urol..

[29] Barclay ME, Lyratzopoulos G, Greenberg DC, Abel GA (2018). Missing data and chance variation in public reporting of cancer stage at diagnosis: cross-sectional analysis of population-based data in England. Cancer Epidemiol..

[30] Luo Q, Egger S, Yu XQ, Smith DP, O’Connell DL (2017). Validity of using multiple imputation for “unknown” stage at diagnosis in population-based cancer registry data. PLoS ONE.

[31] Eisemann N, Waldmann A, Katalinic A (2011). Imputation of missing values of tumour stage in population-based cancer registration. BMC Med. Res. Methodol..

[32] He Y, Yucel R, Zaslavsky AM (2008). Misreporting, missing data, and multiple imputation: improving accuracy of Cancer Registry databases. Chance.

[33] Syriopoulou E, Bower H, Andersson TM, Lambert PC, Rutherford MJ (2017). Estimating the impact of a cancer diagnosis on life expectancy by socio-economic group for a range of cancer types in England. Br. J. Cancer.

[34] Quaife SL, Forbes LJ, Ramirez AJ, Brain KE, Donnelly C, Simon AE (2014). Recognition of cancer warning signs and anticipated delay in help-seeking in a population sample of adults in the UK. Br. J. Cancer.

[35] Niksic M, Rachet B, Duffy SW, Quaresma M, Møller H, Forbes LJ (2016). Is cancer survival associated with cancer symptom awareness and barriers to seeking medical help in England? An ecological study. Br. J. Cancer.

[36] Koo MM, Hamilton W, Walter FM, Rubin GP, Lyratzopoulos G (2018). Symptom signatures and diagnostic timeliness in cancer patients: a review of current evidence. Neoplasia.

[37] Koo MM, Swann R, McPhail S, Abel GA, Elliss-Brookes L, Rubin GP (2020). Presenting symptoms of cancer and stage at diagnosis: evidence from a cross-sectional, population-based study. Lancet Oncol..

[38] Crosby, D., Lyons, N., Greenwood, E., Harrison, S., Hiom, S., Moffat, J. et al. A roadmap for the early detection and diagnosis of cancer. *Lancet Oncol*. **21**, 1397–1399 (2020). 10.1016/S1470-2045(20)30593-3 PMC7535618 33031732

