# Supplementary material for: Socio-demographic variation in stage at diagnosis of breast, bladder, colon, endometrial, lung, melanoma, prostate, rectal, renal and ovarian cancer in England and its population impact
Source: Br J Cancer. 2021 Feb 9;124(7):1320–9. doi: 10.1038/s41416-021-01279-z (PMC8007585; doi:10.1038/s41416-021-01279-z)

Supplementary Information file

Text box: MULTIPLE IMPUTATION

Multiple imputation by chained equations was used to produce ten imputed datasets with complete stage and grade data.[1,2] Separately for each cancer site, a binomial indicator of advanced stage (TNM stages III or IV) at diagnosis was imputed using logistic regression based on age (in the same categorical groups as for the main analysis), gender, deprivation group (fifths of IMD 2010 income domain), ethnicity (white/other), region (with pairwise interactions between region and each of age, gender, deprivation, and ethnicity), tumour grade (I,II,III or IV), basis of diagnosis (death certificate only, non-microscopic diagnosis, microscopic diagnosis), survival from diagnosis (indicators for death within 30 days and within 365 days, and Nelson-Aalen estimator of cumulative hazard up to 365 days after diagnosis), and screening status (screen-detected; not screen-detected; no screening programme for this site). Except for melanoma, tumour grade was also imputed using predictive mean matching.[3] Basis of diagnosis and the indicator for death within 30 days were not used when imputing missing stage for melanoma, or when imputing missing stage and grade for endometrial cancer, because for these cancer sites these variables were associated with very few patients.

1. Rubin DB. Multiple Imputation for Nonresponse in Surveys. John Wiley and Sons: New York. 1987.
2. van Buuren S, Boshuizen HC, Knook DL. Multiple imputation of missing blood pressure covariates in survival analysis. Multiple imputation of missing blood pressure covariates in survival analysis. Stat Med. 1999;18(6):681-94.
3. Morris TP, White IR, Royston P. Tuning multiple imputation by predictive mean matching and local residual draws. BMC Med Res Methodol. 2014;14:75. doi: 10.1186/1471-2288-14-75.

Supplementary Information-Table 1. Coefficients and spotlight estimates for the models shown in Figures 1-4. The first super-row cluster ('Overall') relates to main-text Figure 1, whereas all other rows to cancer-site specific outputs from main-text Figures 2-4.

Cancer site	Variable	Variable category	Odds ratio	(95% CI)
Overall	Age	35	1.09	(1.04, 1.13)
Overall	Age	45	1.03	(0.99, 1.07)
Overall	Age	55	0.96	(0.92, 1.00)
Overall	Age	65	Reference	
Overall	Age	75	1.04	(1.01, 1.08)
Overall	Age	85	1.36	(1.31, 1.41)
Overall	Age	95	1.88	(1.81, 1.95)
Overall	Sex	Men	Reference	
Overall	Sex	Women	0.91	(0.89, 0.94)
Overall	Deprivation	Least deprived	Reference	
Overall	Deprivation	2	1.02	(0.99, 1.06)
Overall	Deprivation	3	1.07	(1.04, 1.11)
Overall	Deprivation	4	1.15	(1.11, 1.18)
Overall	Deprivation	Most deprived	1.17	(1.13, 1.21)
Bladder	Age	35	1.50	(1.12, 2.02)
Bladder	Age	45	1.04	(0.79, 1.38)
Bladder	Age	55	0.80	(0.62, 1.04)
Bladder	Age	65	Reference	
Bladder	Age	75	0.81	(0.69, 0.95)
Bladder	Age	85	0.93	(0.77, 1.11)
Bladder	Age	95	1.21	(1.02, 1.43)
Bladder	Sex	Men	Reference	
Bladder	Sex	Women	1.45	(1.31, 1.61)
Bladder	Deprivation	Least deprived	Reference	
Bladder	Deprivation	2	0.98	(0.85, 1.14)
Bladder	Deprivation	3	1.21	(1.04, 1.41)
Bladder	Deprivation	4	1.14	(0.98, 1.32)
Bladder	Deprivation	Most deprived	1.35	(1.16, 1.57)
Breast	Age	35	1.69	(1.55, 1.84)
Breast	Age	45	1.46	(1.34, 1.58)
Breast	Age	55	1.20	(1.08, 1.33)
Breast	Age	65	Reference	
Breast	Age	75	1.75	(1.58, 1.93)
Breast	Age	85	2.11	(1.90, 2.35)
Breast	Age	95	2.20	(1.98, 2.43)
Breast	Deprivation	Least deprived	Reference	
Breast	Deprivation	2	1.06	(0.98, 1.15)
Breast	Deprivation	3	1.10	(1.01, 1.19)
Breast	Deprivation	4	1.34	(1.24, 1.46)
Breast	Deprivation	Most deprived	1.31	(1.20, 1.43)
Breast, adjusted for screen-detection	Age	35	1.10	(1.00, 1.20)
Breast, adjusted for screen-detection	Age	45	1.04	(0.95, 1.13)
Breast, adjusted for screen-detection	Age	55	1.09	(0.98, 1.22)
Breast, adjusted for screen-detection	Age	65	Reference	
Breast, adjusted for screen-detection	Age	75	1.13	(1.02, 1.26)
Breast, adjusted for screen-detection	Age	85	1.17	(1.05, 1.31)
Breast, adjusted for screen-detection	Age	95	1.16	(1.04, 1.29)
Breast, adjusted for screen-detection	Deprivation	Least deprived	Reference	
Breast, adjusted for screen-detection	Deprivation	2	1.07	(0.99, 1.16)
Breast, adjusted for screen-detection	Deprivation	3	1.10	(1.01, 1.19)
Breast, adjusted for screen-detection	Deprivation	4	1.35	(1.24, 1.46)
Breast, adjusted for screen-detection	Deprivation	Most deprived	1.28	(1.17, 1.39)
Colon	Age	35	1.23	(1.11, 1.37)
Colon	Age	45	1.21	(1.07, 1.36)
Colon	Age	55	1.14	(1.01, 1.29)
Colon	Age	65	Reference	
Colon	Age	75	0.80	(0.73, 0.88)

Cancer site	Variable	Variable category	Odds ratio	(95% CI)
Colon	Age	85	1.00	(0.91, 1.10)
Colon	Age	95	1.41	(1.29, 1.55)
Colon	Sex	Men	Reference	
Colon	Sex	Women	1.06	(1.00, 1.11)
Colon	Deprivation	Least deprived	Reference	
Colon	Deprivation	2	1.02	(0.94, 1.10)
Colon	Deprivation	3	1.06	(0.98, 1.15)
Colon	Deprivation	4	1.12	(1.03, 1.21)
Colon	Deprivation	Most deprived	1.16	(1.07, 1.27)
Colon, adjusted for screen-detection	Age	35	0.92	(0.82, 1.03)
Colon, adjusted for screen-detection	Age	45	0.91	(0.81, 1.04)
Colon, adjusted for screen-detection	Age	55	0.88	(0.77, 1.00)
Colon, adjusted for screen-detection	Age	65	Reference	
Colon, adjusted for screen-detection	Age	75	0.64	(0.58, 0.70)
Colon, adjusted for screen-detection	Age	85	0.72	(0.65, 0.80)
Colon, adjusted for screen-detection	Age	95	0.99	(0.90, 1.10)
Colon, adjusted for screen-detection	Sex	Men	Reference	
Colon, adjusted for screen-detection	Sex	Women	1.04	(0.98, 1.09)
Colon, adjusted for screen-detection	Deprivation	Least deprived	Reference	
Colon, adjusted for screen-detection	Deprivation	2	1.03	(0.95, 1.11)
Colon, adjusted for screen-detection	Deprivation	3	1.06	(0.98, 1.15)
Colon, adjusted for screen-detection	Deprivation	4	1.10	(1.02, 1.20)
Colon, adjusted for screen-detection	Deprivation	Most deprived	1.15	(1.05, 1.25)
Rectal	Age	35	1.74	(1.49, 2.03)
Rectal	Age	45	1.44	(1.23, 1.69)
Rectal	Age	55	1.30	(1.11, 1.52)
Rectal	Age	65	Reference	
Rectal	Age	75	1.01	(0.89, 1.15)
Rectal	Age	85	1.19	(1.04, 1.36)
Rectal	Age	95	1.43	(1.24, 1.64)
Rectal	Sex	Men	Reference	
Rectal	Sex	Women	0.96	(0.89, 1.04)
Rectal	Deprivation	Least deprived	Reference	
Rectal	Deprivation	2	1.05	(0.94, 1.18)
Rectal	Deprivation	3	1.06	(0.95, 1.20)
Rectal	Deprivation	4	1.15	(1.02, 1.30)
Rectal	Deprivation	Most deprived	1.34	(1.19, 1.52)
Rectal, adjusted for screen-detection	Age	35	1.38	(1.18, 1.61)
Rectal, adjusted for screen-detection	Age	45	1.16	(0.98, 1.36)
Rectal, adjusted for screen-detection	Age	55	1.05	(0.89, 1.23)
Rectal, adjusted for screen-detection	Age	65	Reference	
Rectal, adjusted for screen-detection	Age	75	0.85	(0.75, 0.97)
Rectal, adjusted for screen-detection	Age	85	0.92	(0.80, 1.07)
Rectal, adjusted for screen-detection	Age	95	1.08	(0.93, 1.25)
Rectal, adjusted for screen-detection	Sex	Men	Reference	
Rectal, adjusted for screen-detection	Sex	Women	0.94	(0.87, 1.02)
Rectal, adjusted for screen-detection	Deprivation	Least deprived	Reference	
Rectal, adjusted for screen-detection	Deprivation	2	1.05	(0.94, 1.17)
Rectal, adjusted for screen-detection	Deprivation	3	1.06	(0.94, 1.19)
Rectal, adjusted for screen-detection	Deprivation	4	1.13	(1.00, 1.27)
Rectal, adjusted for screen-detection	Deprivation	Most deprived	1.31	(1.15, 1.48)
Endometrial	Age	35	0.90	(0.66, 1.22)
Endometrial	Age	45	1.44	(1.11, 1.87)
Endometrial	Age	55	0.87	(0.68, 1.12)
Endometrial	Age	65	Reference	
Endometrial	Age	75	1.20	(0.96, 1.49)
Endometrial	Age	85	1.61	(1.29, 2.01)
Endometrial	Age	95	2.28	(1.80, 2.89)
Endometrial	Deprivation	Least deprived	Reference	
Endometrial	Deprivation	2	1.08	(0.90, 1.31)
Endometrial	Deprivation	3	1.06	(0.88, 1.29)
Endometrial	Deprivation	4	1.15	(0.95, 1.40)
Endometrial	Deprivation	Most deprived	1.15	(0.94, 1.41)

Cancer site	Variable	Variable category	Odds ratio	(95% CI)
Lung	Age	35	1.42	(1.24, 1.63)
Lung	Age	45	1.44	(1.25, 1.65)
Lung	Age	55	1.14	(1.02, 1.28)
Lung	Age	65	Reference	
Lung	Age	75	0.86	(0.79, 0.93)
Lung	Age	85	0.92	(0.85, 1.00)
Lung	Age	95	1.06	(0.97, 1.16)
Lung	Sex	Men	Reference	
Lung	Sex	Women	0.81	(0.77, 0.85)
Lung	Deprivation	Least deprived	Reference	
Lung	Deprivation	2	0.96	(0.88, 1.04)
Lung	Deprivation	3	1.01	(0.93, 1.09)
Lung	Deprivation	4	0.97	(0.89, 1.05)
Lung	Deprivation	Most deprived	0.90	(0.84, 0.98)
Melanoma	Age	35	0.89	(0.73, 1.08)
Melanoma	Age	45	0.91	(0.74, 1.12)
Melanoma	Age	55	1.05	(0.81, 1.34)
Melanoma	Age	65	Reference	
Melanoma	Age	75	1.14	(0.92, 1.42)
Melanoma	Age	85	1.37	(1.09, 1.72)
Melanoma	Age	95	1.63	(1.31, 2.04)
Melanoma	Sex	Men	Reference	
Melanoma	Sex	Women	0.72	(0.64, 0.81)
Melanoma	Deprivation	Least deprived	Reference	
Melanoma	Deprivation	2	0.92	(0.78, 1.09)
Melanoma	Deprivation	3	1.03	(0.86, 1.23)
Melanoma	Deprivation	4	1.40	(1.17, 1.68)
Melanoma	Deprivation	Most deprived	1.49	(1.22, 1.83)
Ovarian	Age	35	0.54	(0.42, 0.70)
Ovarian	Age	45	0.73	(0.57, 0.94)
Ovarian	Age	55	0.76	(0.57, 1.01)
Ovarian	Age	65	Reference	
Ovarian	Age	75	1.12	(0.84, 1.48)
Ovarian	Age	85	1.47	(1.10, 1.95)
Ovarian	Age	95	2.05	(1.47, 2.85)
Ovarian	Deprivation	Least deprived	Reference	
Ovarian	Deprivation	2	1.09	(0.88, 1.36)
Ovarian	Deprivation	3	1.28	(1.02, 1.61)
Ovarian	Deprivation	4	1.23	(0.97, 1.56)
Ovarian	Deprivation	Most deprived	1.29	(1.00, 1.66)
Prostate	Age	35	0.48	(0.32, 0.73)
Prostate	Age	45	0.59	(0.49, 0.70)
Prostate	Age	55	0.77	(0.69, 0.86)
Prostate	Age	65	Reference	
Prostate	Age	75	0.95	(0.89, 1.02)
Prostate	Age	85	1.82	(1.69, 1.95)
Prostate	Age	95	4.26	(3.93, 4.63)
Prostate	Deprivation	Least deprived	Reference	
Prostate	Deprivation	2	1.04	(0.98, 1.11)
Prostate	Deprivation	3	1.05	(0.99, 1.11)
Prostate	Deprivation	4	1.15	(1.07, 1.22)
Prostate	Deprivation	Most deprived	1.15	(1.08, 1.23)
Renal	Age	35	0.62	(0.53, 0.73)
Renal	Age	45	0.76	(0.65, 0.89)
Renal	Age	55	1.01	(0.85, 1.20)
Renal	Age	65	Reference	
Renal	Age	75	1.00	(0.86, 1.16)
Renal	Age	85	1.16	(0.99, 1.35)
Renal	Age	95	1.38	(1.17, 1.63)
Renal	Sex	Men	Reference	
Renal	Sex	Women	0.78	(0.71, 0.85)
Renal	Deprivation	Least deprived	Reference	
Renal	Deprivation	2	0.93	(0.82, 1.06)

Cancer site	Variable	Variable category	Odds ratio	(95% CI)
Renal	Deprivation	3	1.02	(0.90, 1.17)
Renal	Deprivation	4	0.92	(0.81, 1.06)
Renal	Deprivation	Most deprived	1.02	(0.89, 1.16)

Supplementary Information-Table 2. Estimated impact of removing ‘older age’ and income deprivation inequalities, based on a model adjusting for morphology and screening where appropriate

Cancer	Impact of…	Total diagnosis	Stage 3/4 cancers	(95% CI)	Reduction	(% of total diagnosis)
All combined	Observed	202001	86435	(82637, 90233)		
	Removing ‘older age’ disparities		82180	(78397, 85964)	4255	-2.1%
	Removing deprivation disparities		84050	(80378, 87723)	2385	-1.2%
	Both combined		79817	(76165, 83469)	6618	-3.3%
Colon	Observed	23452	13267	(12778, 13756)		
	Removing ‘older age’ disparities		13193	(12705, 13682)	74	-0.3%
	Removing deprivation disparities		12938	(12457, 13419)	329	-1.4%
	Both combined		12863	(12384, 13343)	404	-1.7%
Rectal	Observed	11279	6542	(6202, 6882)		
	Removing ‘older age’ disparities		6527	(6191, 6863)	15	-0.1%
	Removing deprivation disparities		6298	(5957, 6640)	244	-2.2%
	Both combined		6283	(5947, 6620)	259	-2.3%
Lung	Observed	38086	28256	(27639, 28873)		
	Removing ‘older age’ disparities		28243	(27629, 28856)	13	-0.0%
	Removing deprivation disparities		28248	(27622, 28874)	8	-0.0%
	Both combined		28235	(27612, 28857)	21	-0.1%
Melanoma	Observed	12970	1232	(1020, 1444)		
	Removing ‘older age’ disparities		1086	(890, 1283)	146	-1.1%
	Removing deprivation disparities		1119	(934, 1304)	113	-0.9%
	Both combined		984	(813, 1156)	248	-1.9%
Breast	Observed	45432	7136	(6577, 7695)		
	Removing ‘older age’ disparities		6871	(6314, 7429)	265	-0.6%
	Removing deprivation disparities		6433	(5919, 6946)	703	-1.5%
	Both combined		6186	(5677, 6695)	950	-2.1%
Endometrial	Observed	7316	1560	(1322, 1798)		
	Removing ‘older age’ disparities		1353	(1127, 1580)	207	-2.8%
	Removing deprivation disparities		1480	(1251, 1709)	80	-1.1%
	Both combined		1278	(1061, 1495)	282	-3.9%
Ovarian	Observed	5002	3602	(3392, 3812)		
	Removing ‘older age’ disparities		3462	(3233, 3691)	140	-2.8%
	Removing deprivation disparities		3497	(3283, 3712)	104	-2.1%
	Both combined		3347	(3113, 3581)	254	-5.1%
Prostate	Observed	40959	18572	(18013, 19131)		
	Removing ‘older age’ disparities		15449	(14888, 16011)	3123	-7.6%
	Removing deprivation disparities		17981	(17458, 18504)	591	-1.4%
	Both combined		14854	(14334, 15375)	3718	-9.1%
Renal	Observed	8933	3845	(3526, 4164)		
	Removing ‘older age’ disparities		3652	(3331, 3973)	193	-2.2%
	Removing deprivation disparities		3829	(3511, 4147)	16	-0.2%
	Both combined		3635	(3315, 3956)	210	-2.3%
Bladder	Observed	8572	2423	(2169, 2677)		
	Removing ‘older age’ disparities		2343	(2089, 2598)	80	-0.9%
	Removing deprivation disparities		2227	(1986, 2468)	196	-2.3%
	Both combined		2150	(1909, 2392)	273	-3.2%

Supplementary Information-Figure 1. Comparison of odds ratios by age at diagnosis from the main analysis using multiple imputation for missing stage (blue) and a complete case analysis (red)

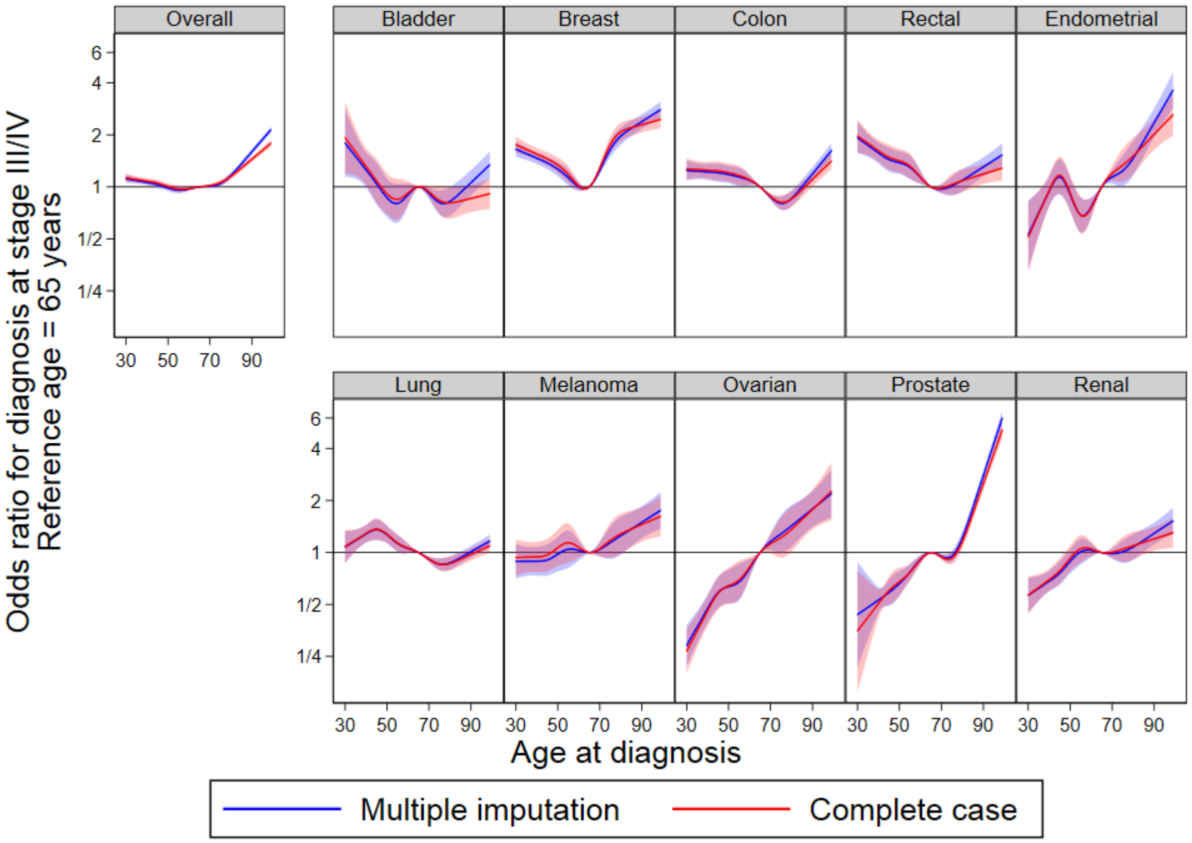

Supplementary Information-Figure 2. Comparison of odds ratios by sex from the main analysis using multiple imputation for missing stage (blue) and a complete case analysis (red)

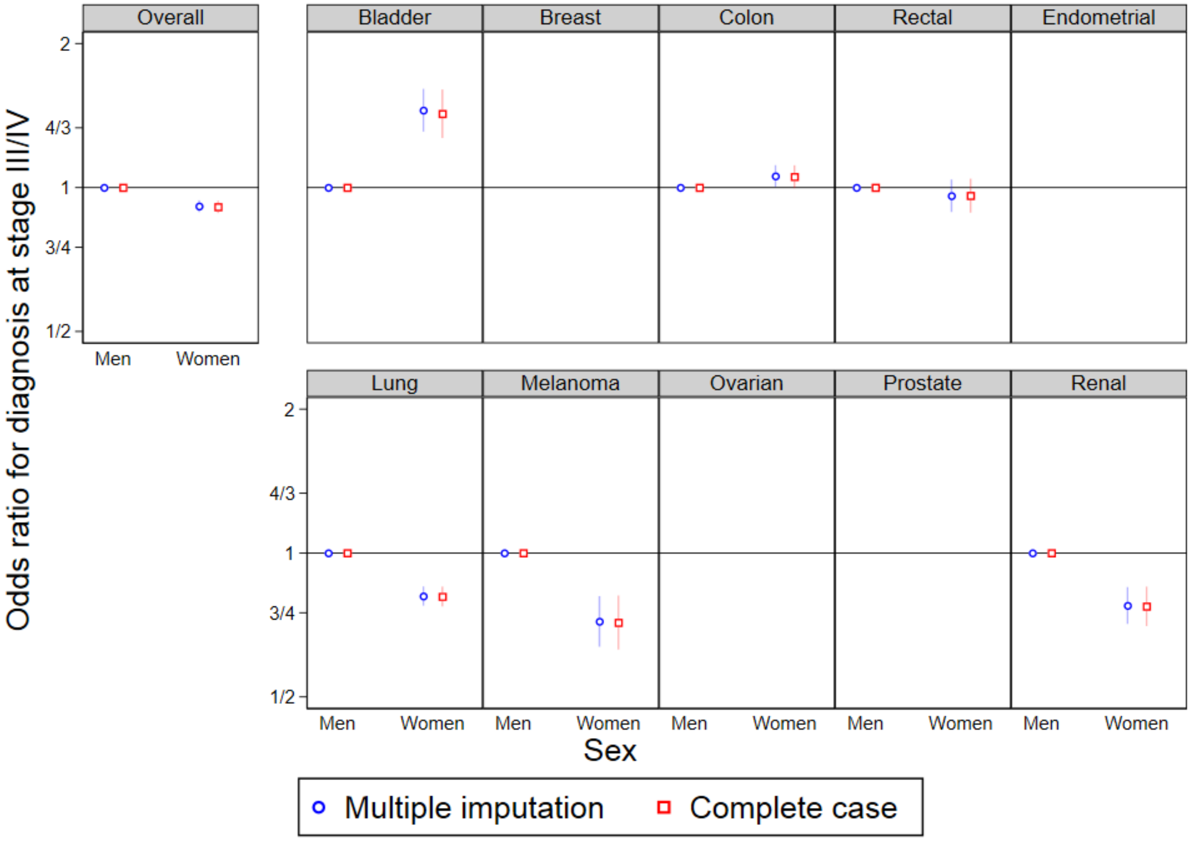

Supplementary Information-Figure 3 Comparison of odds ratios by income deprivation group from the main analysis using multiple imputation for missing stage (blue) and a complete case analysis (red)

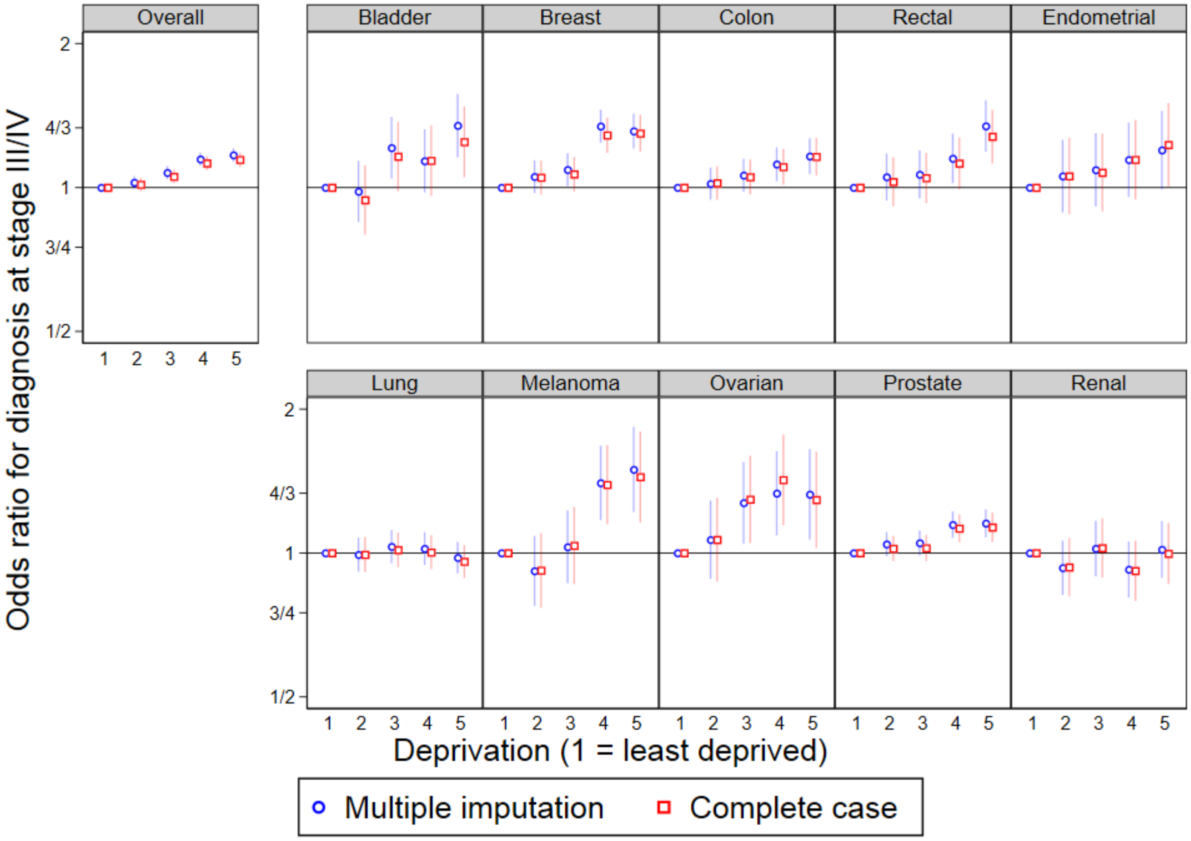

Supplementary Information-Figure 4. Comparison of results by age at diagnosis using three different specifications of 'advanced' stage at diagnosis

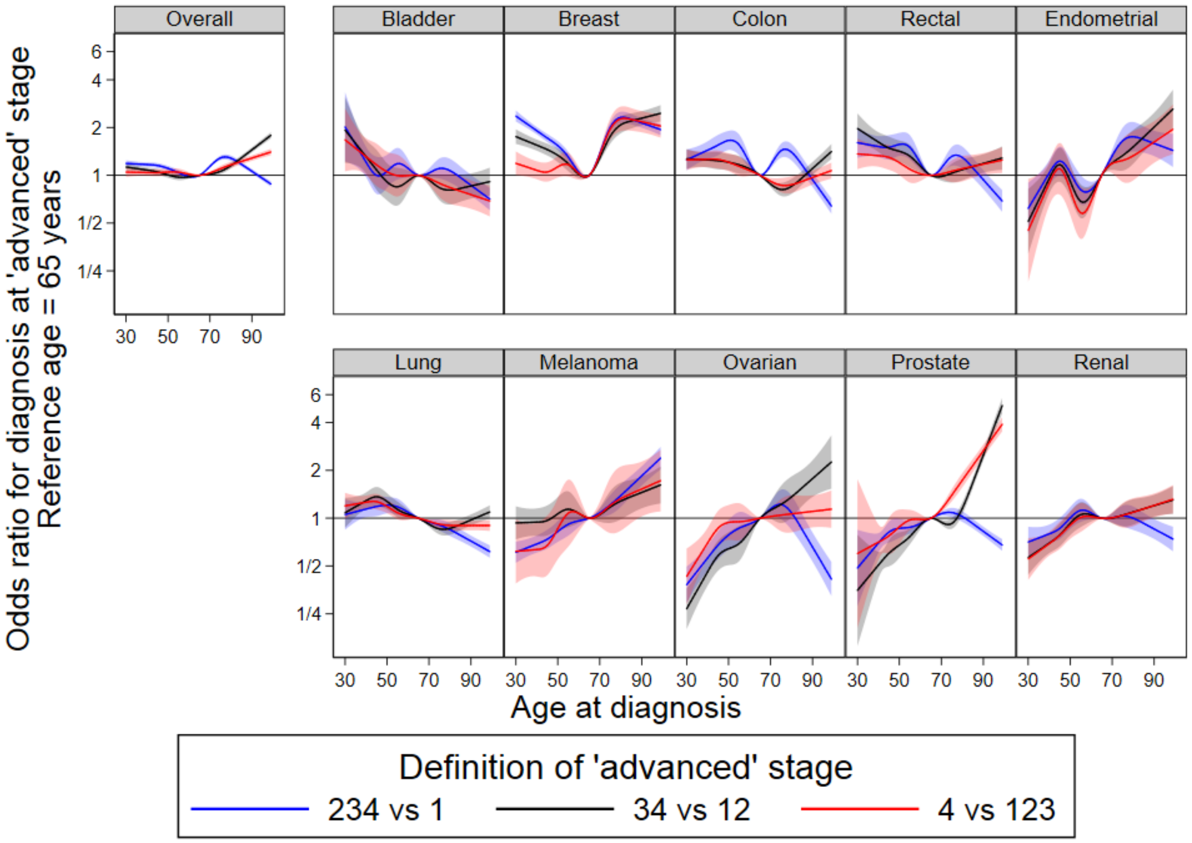

Supplementary Information-Figure 5. Comparison of results by sex using three different specifications of 'advanced' stage at diagnosis.

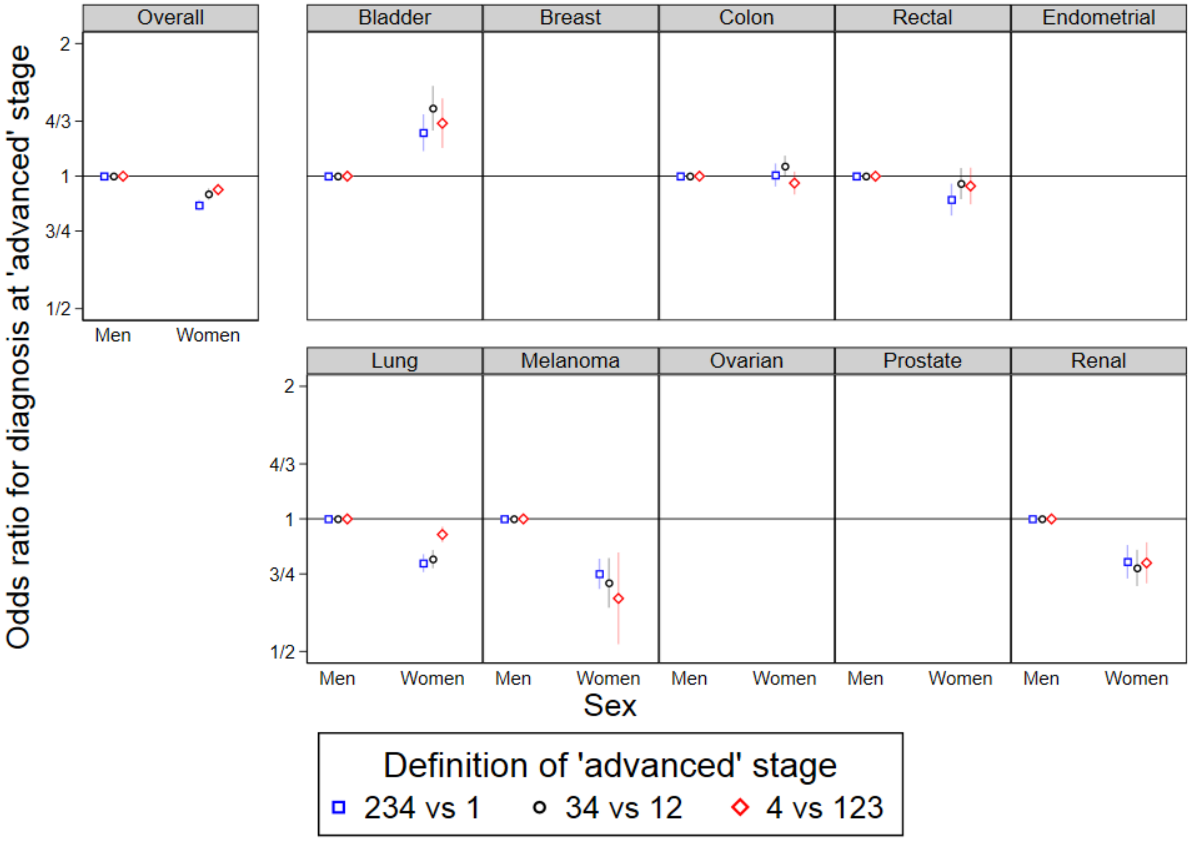

Supplementary Information-Figure 6. Comparison of results by income deprivation using three different specifications of 'advanced' stage at diagnosis.

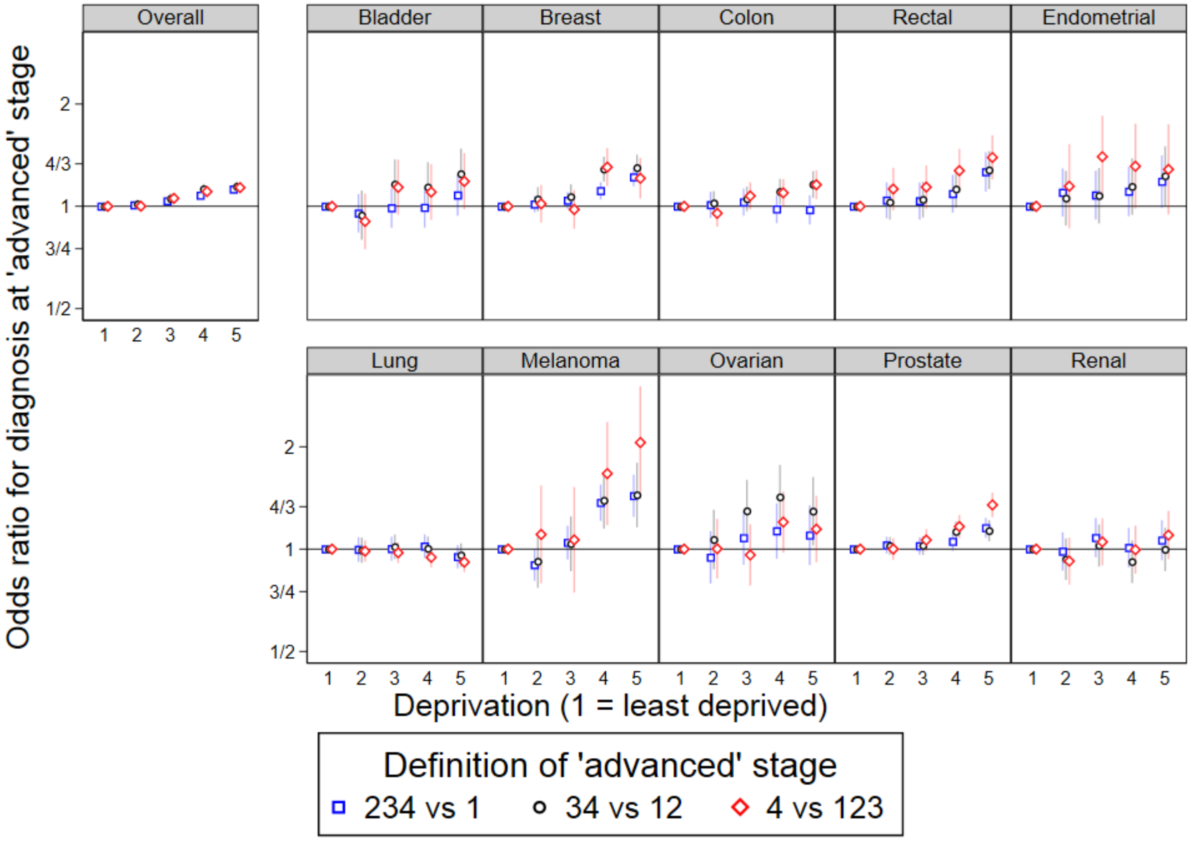

Supplement: Supplementary file 1 — Supplementary online material [file 41416_2021_1279_MOESM1_ESM.pdf]
